# Service use patterns in community mental health outreach: A sequence analysis of the first 12-month longitudinal data

**DOI:** 10.1371/journal.pone.0332437

**Published:** 2025-09-11

**Authors:** Mai Iwanaga, Kaori Usui, Sayaka Sato, Kiyoaki Nakanishi, Erisa Nishiuchi, Michiyo Shimodaira, Yugan So, Sosei Yamaguchi, Chiyo Fujii

**Affiliations:** Department of Community Mental Health & Law, National Institute of Mental Health, National Center of Neurology and Psychiatry, Tokyo, Japan; Khyber Medical University, PAKISTAN

## Abstract

**Aims:**

Community mental health outreach teams offer a range of individualized services tailored to meet the diverse needs of clients. This study aimed to identify service use patterns within community mental health outreach programs using 12-month longitudinal data.

**Methods:**

Data from users of the Tokorozawa City mental health outreach service in Japan were analyzed. Service trajectories over a 365-d period were mapped and categorized by primary service type each month. These trajectories were then analyzed using state sequence and clustering methods. The services included 10 categories (i.e., family support and psychiatric symptom management) and cases of no support. We compared the demographic characteristics (e.g., sex, age, living situation, and diagnosis) and reasons for service initiation (e.g., symptom-related life problems, treatment interruption, or untreated conditions) between clusters.

**Results:**

The service use trajectories of 70 participants were divided into four clusters. Cluster 1 had a high proportion of psychiatric symptom management services (n = 25). Cluster 2 focused primarily on family support (n = 11). Cluster 3 offered a mix of diverse services (n = 21). Cluster 4 involved inter-agency collaboration and early termination of services (n = 13). Significant differences were found between clusters in terms of sex and some reasons for service initiation.

**Conclusions:**

While community mental health outreach programs deliver a broad range of personalized services, we identified four distinct service use patterns over the first 12 months. Given that demographic and clinical characteristics may vary across these patterns, further research with a larger dataset is needed.

## Introduction

Community mental health outreach teams provide services to address a wide range of client needs. These clients often face multiple concurrent issues, such as severe mental illness, family problems, untreated mental health conditions, interrupted treatment, social withdrawal, and challenges in seeking help. Outreach teams are typically composed of multidisciplinary professionals [1,2]. Although each staff member brings their own professional expertise, the team is not bound by a single approach [1–3]. Their role includes connecting individuals to existing medical and social resources within the community, as well as addressing psychiatric and physical symptoms, providing family support, and assisting with daily living tasks. Additionally, these teams offer a broad range of services, including crisis intervention, support with employment or education, and help with interpersonal relationships. Systematic reviews have demonstrated the effectiveness of community mental health outreach in improving clinical and social outcomes, such as psychiatric symptom reduction, enhanced social functioning, and decreased hospitalization rate) [4–6]. Community mental health outreach plays a critical role in supporting individuals with diverse mental health challenges while they continue living in the community.

The diverse types of clients and services in community mental health outreach may result in distinct service use patterns. Service delivery often varies depending on the clinical situation, living environment, and specific needs or challenges of the client at the time services begin. First, the clinical situation includes whether the client has a diagnosis or is engaged in treatment. Globally, many individuals with mental health issues are not connected to mental health services or receiving any form of treatment [7–10]. Even those with a formal diagnosis frequently discontinue their treatment [11]. Factors, such as self-stigma [12,13] and lack of awareness about the need for support [14], often impede service engagement. In such cases, community mental health outreach, which offers decision-making support and accompanies clients to medical appointments, can be particularly valuable. Second, the living situation is another important factor, with family involvement often playing a key role. Families can provide both essential support and exert significant influence on individuals with mental health conditions [15–18]. Therefore, outreach teams frequently support both the client and their family. Third, beyond untreated mental health problems and family dynamics, clients often present with diverse needs at the initiation of service. These may include difficulties with daily living skills, prolonged social withdrawal, recurrent psychiatric hospitalizations, self-harm or harm to others, and social isolation. Consequently, there may be trends in service delivery based on the clinical condition, living situation, and specific client needs at the onset of service.

However, despite the diversity of clients and the broad scope of provided services, the actual patterns of service within community mental health outreach remain insufficiently understood. Previous studies have assessed the amount of provided service by service category. In Japan, a municipality-led outreach team was established in 2015 in Tokorozawa City to support a wide range of individuals with untreated mental health problems, including youth, socially withdrawn persons, and those reluctant to seek help. A study focusing on this team has reported the service intensity of community mental health outreach among people with untreated mental health problems [19]. Other studies have described functioning level-related differences in service provision [20] and longitudinal changes in service frequency during the first year of service [21]. Related studies on Intensive Case Management (ICM) and Assertive Community Treatment (ACT) have identified at least five core services in ICM through network analysis [22], tracked category-specific support in the first year of ICM [23], examined trajectories of staff–client contact in ACT over 48 months [24], and followed Youth Flexible ACT teams over 18 months [25].

Taken together, these studies revealed that service components could be categorized, that service intensity varies across categories, and it changes over time. Yet no study has clarified service use patterns in community mental health outreach while considering both temporal changes and the complexity of real-world care. Sequence clustering analysis may be useful for this purpose. For example, Golay et al. (2022) applied this method in a six-year ICM study, clustering trajectories based on service states such as “no service,” “hospitalization,” “ICM ambulatory care,” and “death” [26]. Although not categorized by service type, this approach demonstrates the potential of sequence clustering in revealing service use patterns in community mental health outreach.

To address this gap, we conducted a study with the aim of identifying service use patterns focusing on both the provided service types and the service delivery trajectory over time in community mental health outreach using state sequence and clustering methods. By analyzing the first 12 months of outreach data for each client, we sought to clarify the types of service trajectories individuals with different initial conditions follow upon initiating service use.

## Materials and methods

### Study design and participants

This retrospective cohort study examined service use patterns based on data from the Tokorozawa City mental health outreach program in Japan. In this outreach team, multidisciplinary professionals provided individualized services tailored to the unique needs of service users. Although the team helped users locate and utilize medical and social resources in the community, some participants still struggled to connect with those services. In such cases, the team directly supported their life in the community, sometimes resolving issues without external resources. Service locations included telephone consultations, visits to the health center where the outreach team was based, and services provided outside the health center, such as at users’ homes or schools. The service users were community residents with a range of mental health issues, including untreated or undiagnosed conditions and severe mental illnesses [19]. Our previous research demonstrated significant improvements in social functioning among users of this outreach team during the first year of service [27]. Participants in this study were newly enrolled users between November 1, 2018, and June 30, 2023. We analyzed the first 365 d of service data for each participant, as well as their clinical data at the start of service. Data were accessed on July 29, 2024, for research purposes. The data was downloaded by the personal information manager (KN) and processed to ensure individuals could not be identified. The fully anonymized data was analyzed by MI. No exclusion criteria were applied. This study was approved by the Research Ethics Committee of the National Center of Neurology and Psychiatry (no. A2023-024). All data were obtained by reading and transcribing existing clinical records from the outreach team’s clinical activities. Since this study did not involve any invasion, intervention, or procurement of new information from the participants for research purposes, informed consent was not required, according to the Ethical Guidelines for Medical and Biological Research Involving Human Subjects in Japan. Instead, prior to the initiation of the study, a public notice document was created to inform participants about the intended use of service record data related to their background characteristics and services provided. The public notice document was approved by the Ethics Committee. The digital version was posted on the official webpage of the Research Ethics Committee of the National Center of Neurology and Psychiatry, and the paper version was posted on the bulletin board in the office of the outreach team. All individuals were given the opportunity to refuse participation.

### Measures

#### Primary service type per month.

We accessed service records from November 1, 2018, to June 30, 2024, and utilized the first 365 d of data for each participant in this study. The service records detailed the time spent on 10 categories of services: 1) assistance with daily living tasks, 2) family support, 3) psychiatric symptom management, 4) physical health services, 5) crisis intervention, 6) consultation on medical treatment, 7) services related to employment or school attendance, 8) social services, 9) interpersonal relationship support, and 10) other services. The “other services” category frequently included activities, such as liaison, coordination, and information sharing, as well as meetings with professionals outside the outreach team regarding client support. Monthly, the total time spent on each service type was calculated, and the “primary service type” for each month was defined as the service with the highest number of minutes recorded. Additionally, the category “no support” was used when outreach services were interrupted or terminated.

#### Demographic and clinical characteristics.

The following variables were obtained from assessment records at the start of outreach services: age, sex, living situation, psychiatric consultation history, diagnosis according to the International Classification of Diseases, 10th Revision (ICD-10), and any hospitalizations within the past 12 months.

#### Reasons for outreach introduction.

We collected the following data to assess the reasons why community mental health outreach services were deemed necessary upon a user’s enrollment:

1. Life problems caused by psychiatric symptoms
  A. Serious problems in fulfilling social roles
  B. Serious problems in carrying out tasks
  C. Social withdrawal (defined as remaining at home for over 6 months without attending work or school and having minimal contact with individuals outside the family due to psychiatric symptoms)
2. Interruption of treatment
3. Lack of prior treatment
4. Lack of knowledge or understanding of their illness or the need for treatment
5. History of psychiatric emergencies, hospitalizations, or at least two discharges in the past year
6. Behavior, such as self-harm, harm to others, or disruptive behavior
7. Long-term psychiatric inpatients requiring housing and other adjustments for discharge
8. Social isolation

### Statistical analysis

Prior to analysis, we performed a data cleaning procedure to ensure the completeness and consistency of the service dataset. The raw service data consisted of electronic records in which we marked each service episode with its start and end time as well as its service category. The outreach staff entered these records, including the service category coding, immediately after providing each service in clinical practice. From this dataset, we extracted 12 months of data for each participant in this study, defined as newly enrolled users between November 1, 2018, and June 30, 2023, starting from the date of outreach service initiation. For each service episode, we calculated the duration from the recorded start and end times. Using the service initiation date as the baseline, we aggregated the total number of service episodes and total minutes of service for each service category within each month from month 1–12. We coded months with no recorded services as “no support,” and finalized the dataset accordingly.

For the analysis, we used the first 12 months of service data for each participant, along with clinical data collected at the start of outreach service use. First, a sequence clustering analysis was conducted. The service type with the longest duration each month was designated as the “primary service type.” Only the first 12 months following service initiation were included in the sequence analysis; service episodes beyond this period were excluded. We then analyzed the 12-month sequences of primary service types using discrete sequential state/event analysis [28] and grouped the sequences into clusters. Ward’s agglomerative method was applied to the dissimilarity matrix to group similar individual trajectories, with a dendrogram used to determine the number of clusters. Sequence index plots were created to visualize the monthly trajectories of primary service types within each cluster. Status proportion plots were also generated to display the proportion of each service type per cluster, with a maximum value set at 1.0. Second, to identify the characteristics of each cluster, we conducted exploratory comparisons of demographic and clinical characteristics, as well as reasons for outreach service initiation, across clusters using chi-square tests. Since the clinical data contained missing values, we excluded cases with missing data from each chi-square test. The number of missing values is indicated as “Missing” in the results table. Additionally, to examine the service patterns within each cluster, we visualized the transition of service minutes across different service types. All analyses were performed using R statistical software (version 4.0.3, The R Foundation for Statistical Computing, Vienna, Austria). The packages “readxl”, “dplyr”, and “tidyr” were used for data preprocessing, while the “TraMineR” and “cluster” packages were used for state sequence clustering analysis.

## Results

**Fig 1:**
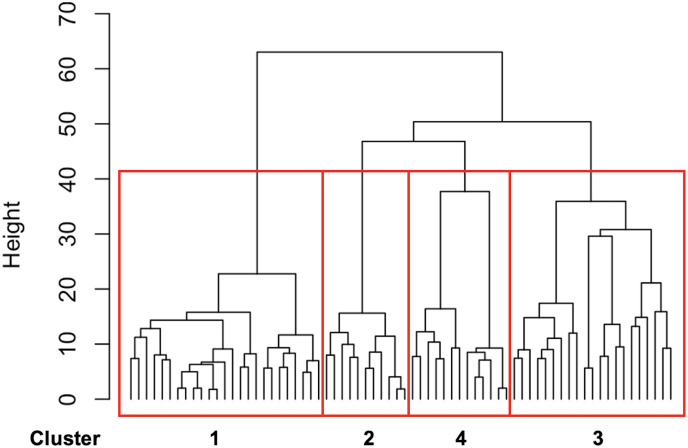
Dendrogram illustrated by hierarchical clustering.

**Fig 2:**
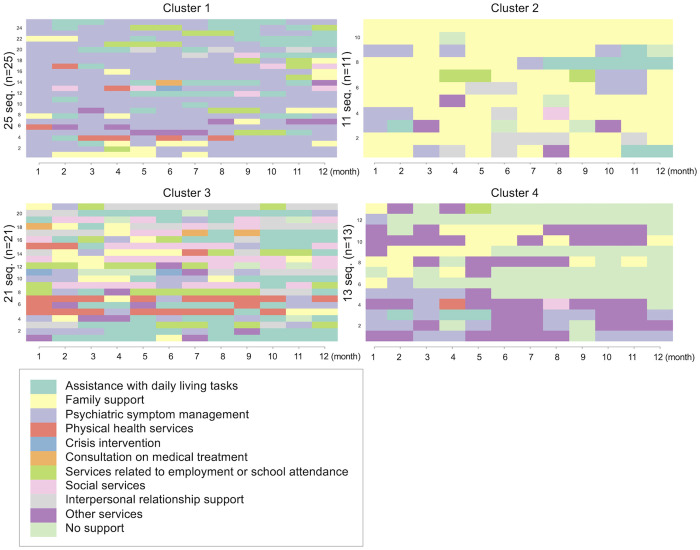
Sequence index plot.

**Fig 3:**
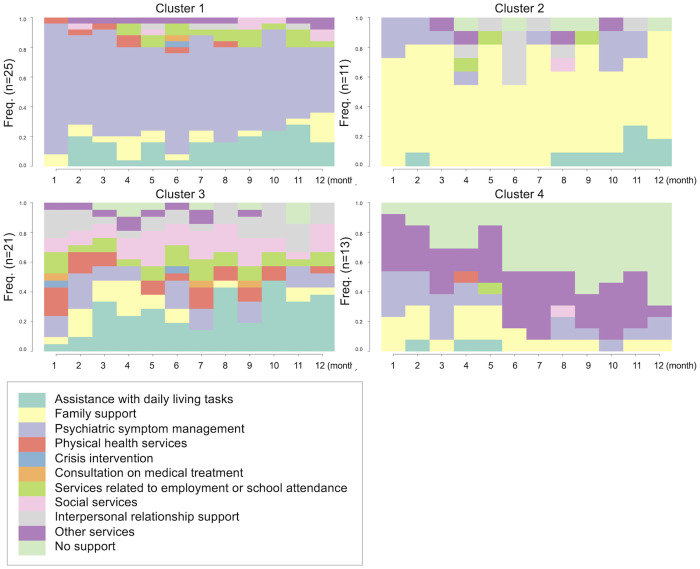
Status proportion plot.

A total of 70 participants with 12 months of service data were included in this study. The mean age was 36.2 years (SD = 19.0), and 46% of the participants were male. Sequence clustering analysis based on the 12-month trajectories of primary service types revealed four distinct clusters, as shown by the dendrogram (Fig 1). Fig 2 presents the sequence index plot, and Fig 3 displays the status proportion plot of the clusters. Based on these visualizations, cluster 1 (n = 25; 36%) comprised users who predominantly received services for psychiatric symptoms during the first 12 months of outreach. Cluster 2 (n = 11; 16%) consisted of users who primarily received family support services. Cluster 3 (n = 21; 30%) included users who received a variety of services. Cluster 4 (n = 13; 19%) represented users whose services were interrupted or ended before reaching 12 months.

Table 1 summarizes the participants’ characteristics at the start of community mental health outreach service use. There was a significant difference in the proportion of male across the clusters (cluster 1, 68%; cluster 2, 27%; cluster 3, 33%; cluster 4, 39%; p = 0.04). The mean age was 40.4 years (SD = 19.5) in cluster 1, 28.4 years (SD = 17.2) in cluster 2, 36.0 years (SD = 18.7) in cluster 3, and 35.2 years (SD = 19.4) in cluster 4. Table 2 shows the differences between clusters regarding the reasons for outreach service initiation. Significant differences were observed for users with interrupted treatment (cluster 1, 56%; cluster 2, 27%; cluster 3, 0%; cluster 4, 23%; p < 0.01) and untreated users (cluster 1, 4%; cluster 2, 18%; cluster 3, 28%; cluster 4, 23%; p = 0.04). No significant differences were found for other variables.

**Table 1:** Participants’ characteristics at the start of community mental health outreach service use in Japan (n = 70).

	Cluster 1 (n = 25)		Cluster 2 (n = 11)		Cluster 3 (n = 21)		Cluster 4 (n = 13)		p-value^a^
	n	%	n	%	n	%	n	%	
**Sex**									0.04*
Male	17	68%	3	27%	7	33%	5	39%	
Female	8	32%	8	73%	14	67%	8	62%	
**Living situation**									0.21
Living alone	7	28%	0	0%	1	5%	3	23%	
Living with family	17	68%	11	100%	19	91%	9	69%	
Missing	1	4%	0	0%	1	5%	1	8%	
**Psychiatric consultation history**									0.05
Have	24	96%	8	73%	13	62%	9	69%	
Not have	0	0%	3	27%	8	38%	4	31%	
Missing	1	4%	0	0%	0	0%	0	0%	
**Diagnosis according to the ICD-10 classification**									0.07
Undiagnosed	2	8%	3	27%	8	38%	5	39%	
Diagnosed	23	92%	8	73%	13	62%	8	62%	
F0: Mental disorders due to known physiological conditions	1	4%	0	0%	2	10%	0	0%	
F1: Mental and behavioral disorders due to psychoactive substance use	1	4%	0	0%	0	0%	0	0%	
F2: Schizophrenia, schizotypal, delusional, and other non-mood psychotic disorders	9	36%	1	9%	4	19%	3	23%	
F30–F31: Mood [affective] disorders	1	4%	0	0%	1	5%	0	0%	
F32–F39: Mood [affective] disorders	4	16%	2	18%	1	5%	2	15%	
F4: Anxiety, dissociative, stress-related, somatoform, and other nonpsychotic mental disorders	1	4%	2	18%	3	14%	2	15%	
F5: Behavioral syndromes associated with physiological disturbances and physical factors	0	0%	0	0%	0	0%	0	0%	
F6: Disorders of adult personality and behavior	1	4%	0	0%	0	0%	0	0%	
F7: Intellectual disabilities	0	0%	0	0%	0	0%	1	8%	
F8: Pervasive and specific developmental disorders	3	12%	2	18%	2	10%	0	0%	
F9:	0	0%	1	9%	0	0%	0	0%	
Others	2	8%	0	0%	0	0%	0	0%	
**Hospitalization within the past 12 months**									0.30
Have been hospitalized	8	32%	1	9%	3	14%	4	31%	
Have not been hospitalized	17	68%	10	91%	18	86%	9	69%	

ICD-10: The International Classification of Diseases 10th Revision

^a^Chi-square tests were used.

**Table 2:** Reasons why community mental health outreach service was deemed necessary for the participants at the start of service use (n = 70).

	Cluster 1 (n = 25)		Cluster 2 (n = 11)		Cluster 3 (n = 21)		Cluster 4 (n = 12)		p-value^a^
	n	%	n	%	n	%	n	%	
1. Life problems caused by psychiatric symptoms	25	100%	8	73%	18	86%	10	77%	0.07
A. Serious problems in fulfilling social roles	21	84%	8	73%	18	86%	7	54%	0.13
B. Serious problems in carrying out tasks	21	84%	7	64%	13	62%	6	46%	0.11
C. Social withdrawal	13	52%	4	36%	6	29%	3	23%	0.25
2. Interruption of treatment	14	56%	3	27%	0	0%	3	23%	<0.001*
3. Lack of prior treatment	1	4%	2	18%	8	28%	3	23%	0.04*
4. Lack of knowledge or understanding of their illness or the need for treatment	11	44%	4	36%	7	33%	4	31%	0.83
5. History of psychiatric emergencies, hospitalizations, or at least two discharges in the past year	0	0%	0	0%	1	5%	2	15%	0.14
6. Behavior, such as self-harm, harm to others, or disruptive behavior	12	48%	6	55%	8	38%	5	39%	0.77
7. Long-term psychiatric inpatients requiring housing and other adjustments for discharge	1	4%	0	0%	1	5%	1	8%	0.83
8. Social isolation	11	40%	2	18%	13	62%	7	54%	0.10

^a^Chi-square test were used.

**Fig 4:**
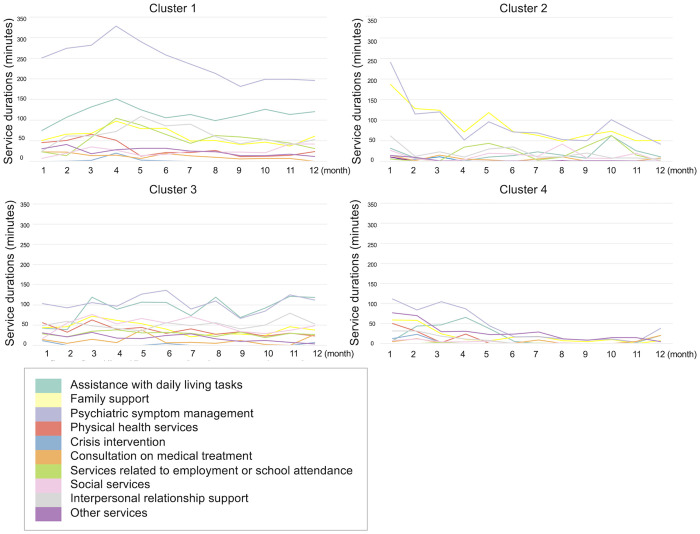
Changes in service time during the first 12 months of community mental health outreach use.

Fig 4 and S1 Table illustrate the changes in service minutes provided during the first 12 months of outreach. Cluster 1 had the highest service duration for psychiatric symptoms, peaking in the fourth month (average: 328 min) before declining. Cluster 2 received similar amounts of family support and psychiatric symptom services, with a gradual decline over time. Cluster 3 maintained a stable level of diverse services, with a notable amount of time spent on assistance with daily living tasks and psychiatric symptom services. Cluster 4 received the least service overall and included a high proportion of “other support” services, such as liaison, coordination, information sharing, and meetings with professionals outside the outreach team.

## Discussion

This study examined the first 12 months of service trajectories for newly enrolled users of community mental health outreach programs. Despite the diversity of services offered, four distinct service use patterns were identified: (1) a high proportion of services focused on psychiatric symptoms (Cluster 1), (2) a high proportion of family support (Cluster 2), (3) diverse types of services provided (Cluster 3), and (4) inter-agency collaboration and/or early service termination (Cluster 4). Significant differences were observed between clusters in terms of sex distribution and the prevalence of users who had interrupted treatment or were untreated at the start of outreach. The amount of service provided and its variation over time were also characteristics of each cluster.

The findings of this study provide new insights into service use patterns in community mental health outreach. Consistent with previous studies conducted in Japan [19–21], “assistance with daily living tasks,” “family support,” and “psychiatric symptom management” accounted for the majority of total service time. However, while “family support” and “psychiatric symptom management” were distinctively divided across clusters, “assistance with daily living tasks” was provided across multiple clusters, indicating its broad relevance for diverse clients. Moreover, whereas earlier research on community mental health outreach and ICM programs reported a decreasing trend in the total amount of support during the first 12 months of service use [21,23], Cluster 3 in this study showed no such decline, suggesting that service provision does not necessarily diminish over time. Previous longitudinal research over a four-year period found that staff–client contact frequency and duration initially spiked, dropped sharply in month 8, and then diverged: contact frequency increased, while contact duration declined slowly [24]. While our study identified clusters by service duration, the trajectories focusing on service frequency might reveal different patterns. Furthermore, unlike prior studies that primarily tracked changes in service amounts by category, the present study identified and examined a distinct cluster characterized by inter-agency collaboration and/or early service termination (Cluster 4), which could not have been captured through simple trajectory analyses of service categories alone. Taken together, this study advances the understanding of service use patterns by addressing both the types of services provided and their trajectories over time. In the following sections, we discuss the characteristics of each cluster in detail.

### Cluster 1: high proportion of services focused on psychiatric symptoms

In Cluster 1, all users experienced life difficulties related to psychiatric symptoms and consistently received services focused on managing these symptoms as their primary service type. This cluster had the longest service durations compared to the others. Notably, there was a high proportion of male and individuals who had discontinued medical treatment at the start of outreach in Cluster 1. Research has shown that even short treatment interruptions (less than 2 weeks) can exacerbate symptoms and nearly double the risk of hospitalization [29]. Moreover, treatment discontinuation itself is often driven by symptom deterioration and noncompliance [30]. Factors, such as self-stigma [12,13] and a lack of awareness of the need for treatment [14], may also contribute to discontinuation, yet these issues cannot be easily addressed by conventional medical care alone [11]. Consequently, it may be crucial for community mental health outreach teams to provide sustained support for psychiatric symptoms, alongside decision-making assistance, accompaniment to medical appointments, and coordination with medical institutions.

### Cluster 2: high proportion of family support

Cluster 2 consisted of users who primarily received family support services alongside services for psychiatric symptoms. All users in this cluster lived with their families, and the majority were female. Previous studies have shown that individuals with mental health issues often seek help from family members [15]. Furthermore, both mental health and the utilization of mental health services are influenced by the mental health status of family members [16,17]. Additionally, earlier research on community outreach programs suggests that reducing the burden on families caring for individuals with mental illness can lead to positive outcomes, including reductions in psychiatric symptoms [18]. The family support provided by outreach teams includes evidence-based interventions aimed at assisting family members [31]. It is likely that family support plays a crucial role in aiding clients, particularly when integrated into the broader outreach strategy.

### Cluster 3: diverse types of services

Cluster 3 was characterized by a diverse range of services. The primary service in this cluster involved assistance with daily living tasks, physical health services and social services, or interpersonal relationship support. This cluster also included cases which the primary service type frequently shifted from month to month. In addition, it had the highest proportion of untreated users. According to the World Mental Health Survey, many individuals with mental health conditions are not engaged with mental health services, despite an increase in service use over recent decades [7–10]. Community mental health outreach programs may serve as the first point of contact for untreated individuals with various issues [19]. The results of this study also showed that, in these cases, outreach programs were not only helping to connect people to existing medical and social services, but were also assisting them with their individual or social struggles, which might shift from day to day. However, in the future, new themes and additional clusters may emerge if a study is conducted with a larger sample size. Cluster 3 did not show a decrease in minutes of service; it remained consistent over time even though the amount of service was initially lower than in other clusters. Further follow-up research is needed to determine how service would transition after the second year of outreach service use.

### Cluster 4: inter-agency collaboration and/or early service termination

Cluster 4 had fewer total service minutes than the other clusters and included many users whose services were interrupted or ended prior to the completion of the 12-month study period. This cluster also had a higher proportion of “other” services, which included liaison, coordination, information sharing, and meetings with professionals outside the outreach team. Since the needs of clients in this cluster appeared to vary widely, the purposes of collaboration with other agencies might have also varied. For instance, if outreach users need specialized treatment, such as medication or hospitalization, transition support was provided to connect them to the appropriate resources. Although no significant differences were found, this cluster had a higher proportion of users with a history of psychiatric emergencies, repeated hospitalizations (at least twice in the past year), and long-term psychiatric inpatients requiring housing or discharge planning than other clusters. Simultaneously, many untreated individuals were included in this cluster. For these cases, the outreach team’s primary role may have been to facilitate connections to existing medical and social resources, addressing complex needs that require inter-agency collaboration. The content of inter-agency collaboration and the differences in characteristics between those who received “other” services (i.e., inter-agency collaboration) and those who terminated services early require further research.

### Strengths and limitations

The strength of this study lies in its ability to visualize the trajectory of outreach service use during the first 12 months and identify distinct service use patterns. This approach enabled us to establish a typology of service users and quantify the proportions within each cluster. The findings offer a foundation for further research into the specific needs of outreach users and the types of support they require. Clinically, the results may assist in the management and tailoring of outreach services as new users are integrated into programs.

However, this study had several limitations. First, the analysis relied on the total amount of service provided per month without accounting for variations in service frequency or duration. For instance, there may be significant differences between receiving one 4-h service session per month versus 1-h sessions spread across 4 weeks. A more detailed analysis of individual service patterns, including the frequency of service, could offer deeper insights into the actual service delivery within each cluster. Furthermore, a mixed methods approach would be useful; the use of both quantitative and qualitative data would show a more comprehensive understanding of the complex support patterns. Second, the sample size of our study was relatively small, as it was based on data from only one community mental health outreach team. This might have limited the statistical power. Further research with larger, more diverse datasets is needed to explore differences in characteristics between clusters. In addition, longer-term follow-up data beyond 12 months should also be collected to examine changes in outreach service use over time.

## Conclusions

Community mental health outreach programs provide a wide range of individualized services. This study identified four distinct patterns of service use over the first 12 months of outreach: (1) a high proportion of services focused on psychiatric symptoms, (2) a high proportion of family support, (3) diverse type of services, and (4) inter-agency collaboration and/or early service termination. The demographic and clinical characteristics of users may vary across these patterns. To better understand these variations and inform more targeted outreach strategies, more in-depth studies using larger data sets and using a mixed methods approach are needed.

## Supporting information

S1 Table Average total service hours (minutes) by service types during first 12-month (n = 70). (DOCX)
