# Supplementary material for: Service use patterns in community mental health outreach: A sequence analysis of the first 12-month longitudinal data
Source: PLoS One. 2025 Sep 11;20(9):e0332437. doi: 10.1371/journal.pone.0332437 (PMC12425182; doi:10.1371/journal.pone.0332437)
Supplement: S1 Table — (DOCX) [file pone.0332437.s001.docx]

S1 Table. Average total service hours (minutes) by service types during first 12-month (n = 70)

|  | Month | | | | | | | | | | | |
| --- | --- | --- | --- | --- | --- | --- | --- | --- | --- | --- | --- | --- |
| Service types | 1 | 2 | 3 | 4 | 5 | 6 | 7 | 8 | 9 | 10 | 11 | 12 |
| **Cluster 1** (n = 25) |  |  |  |  |  |  |  |  |  |  |  |  |
| Assistance with daily living tasks | 74.6 | 106.8 | 132.0 | 151.4 | 125.2 | 105.8 | 113.7 | 98.4 | 111.0 | 126.0 | 113.3 | 120.2 |
| Family support | 50.2 | 66.0 | 67.4 | 97.4 | 79.2 | 80.3 | 49.6 | 50.6 | 40.4 | 46.6 | 36.8 | 60.6 |
| Psychiatric symptom management | 250.9 | 274.2 | 281.9 | 328.1 | 290.5 | 258.3 | 235.8 | 213.2 | 181.6 | 199.2 | 198.8 | 196.2 |
| Physical health services | 45.2 | 50.3 | 65.6 | 51.0 | 12.8 | 21.4 | 21.0 | 26.2 | 11.2 | 11.8 | 14.5 | 23.2 |
| Crisis intervention | 0.2 | 0.6 | 2.4 | 19.8 | 2.6 | 1.0 | 0.0 | 0.0 | 0.0 | 0.0 | 0.0 | 0.0 |
| Consultation on medical treatment | 22.8 | 22.2 | 13.7 | 14.9 | 7.0 | 18.6 | 13.2 | 9.8 | 5.5 | 6.6 | 6.8 | 0.0 |
| Services related to employment or school attendance | 22.8 | 13.6 | 57.8 | 104.6 | 87.7 | 65.5 | 43.6 | 62.7 | 59.3 | 52.8 | 44.2 | 30.7 |
| Social services | 7.0 | 19.2 | 34.8 | 26.8 | 13.8 | 16.6 | 24.8 | 22.2 | 22.4 | 20.6 | 42.1 | 42.0 |
| Interpersonal relationship support | 24.2 | 61.4 | 61.5 | 72.4 | 108.8 | 86.2 | 90.2 | 60.2 | 42.1 | 53.8 | 39.8 | 52.8 |
| Other services | 30.2 | 40.7 | 18.2 | 27.9 | 31.4 | 31.7 | 24.7 | 23.4 | 13.8 | 13.9 | 17.0 | 11.6 |
| **Cluster 2** (n = 11) |  |  |  |  |  |  |  |  |  |  |  |  |
| Assistance with daily living tasks | 31.8 | 8.2 | 0.0 | 1.4 | 10.0 | 13.6 | 23.0 | 15.1 | 7.3 | 62.3 | 25.6 | 10.6 |
| Family support | 186.5 | 128.2 | 124.3 | 70.9 | 118.4 | 72.3 | 63.0 | 48.5 | 63.4 | 72.7 | 50.2 | 50.3 |
| Psychiatric symptom management | 240.2 | 115.0 | 120.2 | 51.4 | 95.6 | 70.9 | 69.0 | 52.7 | 49.7 | 101.2 | 69.9 | 41.6 |
| Physical health services | 5.5 | 3.6 | 10.9 | 0.0 | 0.0 | 0.0 | 0.0 | 0.0 | 0.0 | 0.0 | 0.0 | 0.0 |
| Crisis intervention | 10.5 | 0.0 | 0.5 | 0.0 | 0.0 | 0.0 | 0.0 | 1.8 | 0.0 | 0.0 | 0.0 | 0.0 |
| Consultation on medical treatment | 14.1 | 1.5 | 15.0 | 5.0 | 3.6 | 0.0 | 5.5 | 11.1 | 0.0 | 0.0 | 1.4 | 8.2 |
| Services related to employment or school attendance | 5.9 | 0.0 | 0.9 | 34.5 | 43.6 | 28.4 | 2.9 | 10.0 | 37.3 | 62.7 | 15.5 | 0.9 |
| Social services | 26.4 | 5.5 | 0.5 | 3.8 | 19.5 | 17.9 | 11.9 | 41.8 | 8.2 | 7.0 | 19.7 | 0.0 |
| Interpersonal relationship support | 61.4 | 11.8 | 22.7 | 10.5 | 30.0 | 35.0 | 10.5 | 10.9 | 20.0 | 8.2 | 8.2 | 5.5 |
| Other services | 14.1 | 9.6 | 0.9 | 0.5 | 0.5 | 1.4 | 0.5 | 0.5 | 1.8 | 1.8 | 0.9 | 0.7 |
| **Cluster 3** (n = 21) |  |  |  |  |  |  |  |  |  |  |  |  |
| Assistance with daily living tasks | 42.4 | 38.8 | 119.0 | 89.5 | 106.7 | 106.3 | 73.4 | 119.3 | 69.0 | 92.0 | 121.3 | 118.4 |
| Family support | 43.7 | 46.2 | 72.1 | 61.9 | 53.6 | 40.2 | 21.0 | 28.3 | 28.0 | 24.5 | 46.3 | 38.0 |
| Psychiatric symptom management | 103.5 | 92.8 | 105.8 | 97.0 | 126.5 | 136.3 | 89.7 | 109.6 | 66.6 | 84.4 | 124.9 | 112.1 |
| Physical health services | 55.7 | 32.6 | 62.9 | 39.8 | 44.3 | 29.8 | 40.5 | 27.1 | 33.8 | 22.6 | 30.0 | 23.3 |
| Crisis intervention | 11.4 | 0.0 | 0.0 | 0.0 | 0.0 | 4.5 | 0.0 | 0.0 | 0.0 | 0.0 | 0.0 | 6.7 |
| Consultation on medical treatment | 14.3 | 5.2 | 15.2 | 6.9 | 38.1 | 6.0 | 7.6 | 5.2 | 12.1 | 2.1 | 0.2 | 26.8 |
| Services related to employment or school attendance | 28.6 | 21.5 | 34.8 | 38.3 | 30.7 | 32.2 | 29.8 | 22.1 | 32.6 | 19.3 | 29.6 | 26.9 |
| Social services | 16.9 | 55.3 | 76.4 | 52.9 | 66.2 | 55.4 | 71.0 | 53.7 | 34.6 | 28.9 | 37.3 | 48.3 |
| Interpersonal relationship support | 49.0 | 59.3 | 48.3 | 41.9 | 36.0 | 56.2 | 48.8 | 55.7 | 41.0 | 50.0 | 79.5 | 53.1 |
| Other services |  |  |  |  |  |  |  |  |  |  |  |  |
| **Cluster 4** (n = 12) |  |  |  |  |  |  |  |  |  |  |  |  |
| Assistance with daily living tasks | 6.9 | 43.8 | 46.9 | 65.4 | 36.5 | 5.4 | 0.0 | 0.4 | 0.0 | 0.0 | 2.3 | 20.4 |
| Family support | 58.9 | 57.7 | 24.2 | 10.4 | 7.3 | 15.8 | 17.7 | 6.9 | 5.0 | 10.4 | 0.4 | 6.9 |
| Psychiatric symptom management | 111.2 | 83.8 | 104.6 | 86.9 | 43.5 | 16.9 | 17.3 | 11.2 | 8.8 | 11.2 | 3.5 | 38.1 |
| Physical health services | 50.0 | 30.4 | 2.3 | 23.5 | 0.0 | 0.0 | 0.0 | 0.0 | 0.0 | 0.0 | 0.0 | 0.0 |
| Crisis intervention | 12.3 | 23.1 | 2.3 | 0.0 | 0.0 | 0.0 | 0.0 | 0.0 | 0.0 | 0.0 | 0.0 | 0.0 |
| Consultation on medical treatment | 5.0 | 12.3 | 1.2 | 0.0 | 0.0 | 1.5 | 9.2 | 0.0 | 0.0 | 0.0 | 5.4 | 20.4 |
| Services related to employment or school attendance | 0.0 | 0.0 | 3.1 | 0.0 | 0.8 | 0.0 | 1.5 | 0.0 | 0.4 | 0.0 | 0.0 | 0.0 |
| Social services | 7.7 | 11.5 | 4.2 | 4.2 | 5.8 | 0.8 | 0.0 | 1.9 | 0.0 | 0.4 | 0.0 | 0.8 |
| Interpersonal relationship support | 31.5 | 31.2 | 18.5 | 11.5 | 7.7 | 0.0 | 0.0 | 0.0 | 0.0 | 0.0 | 0.0 | 0.8 |
| Other services | 76.7 | 69.7 | 29.5 | 30.5 | 22.7 | 23.8 | 28.8 | 12.0 | 8.5 | 14.6 | 14.6 | 5.0 |
